# How to Evaluate COVID-19 Vaccine Effectiveness—An Examination of Antibody Production and T-Cell Response

**DOI:** 10.3390/diagnostics12061401

**Published:** 2022-06-06

**Authors:** Yi-Chen Fu, Ying-Shih Su, Ching-Fen Shen, Chao-Min Cheng

**Affiliations:** 1 Institute of Biomedical Engineering, National Tsing Hua University, Hsinchu 300, Taiwan; sandy216621@gmail.com (Y.-C.F.); 109005@w.tmu.edu.tw (Y.-S.S.); 2 Division of Infectious Disease, Department of Internal Medicine, Wan Fang Medical Center, Taipei Medical University, Taipei 110, Taiwan; 3 Department of Internal Medicine, School of Medicine, College of Medicine, Taipei Medical University, Taipei 110, Taiwan; 4 Department of Pediatrics, National Cheng Kung University Hospital, College of Medicine, National Cheng Kung University, Tainan 704, Taiwan

The COVID-19 pandemic has had an enormous impact on individuals, societies, and economies worldwide and has resulted in a significant loss of life worldwide. To ameliorate this situation, various vaccines have been developed, distributed, and administered. These vaccines have had a tremendous positive impact, but, as with all such drugs, effectiveness evaluations can be leveraged to confirm impact and utility, and possibly improve product development. Existing COVID-19 vaccines currently take three different forms: (1) mRNA vaccines such as the Pfizer-BioNTech vaccine and the Moderna vaccine; (2) viral vector vaccines such as the ChAdOx1nCoV-19 (AZ) vaccine and the Janssen (Johnson & Johnson) vaccine; and (3) protein subunit vaccines such as the Novavax vaccine [1,2]. Messenger RNA vaccine contains the genetic code (mRNA) of the spike protein (found on the surface of the SARS-CoV-2 virus), when this genetic material enters the body, subsequently produced spike protein triggers an immune response [3]. Viral vector vaccines also contain genetic information from the SARS-CoV-2 virus, but the genetic material is encapsulated in a different virus (a viral vector) such as adenovirus. Viral vector vaccines similarly produce an immune response to spike protein. Protein subunit vaccines contain a portion of the coronavirus spike protein that the immune system recognizes and uses to establish an immune response to the SARS-CoV-2 virus [4,5,6,7]. Each of these vaccines induces an immune response against the SARS-CoV-2 virus, and a comparative evaluation of the effectiveness of each can provide critical information and promote significant subsequent impact. Vaccine efficacy evaluation can be divided into two parts or types (Figure 1): (1) humoral immunity (antibody production); and (2) cellular immunity (T cell response), both of which will be introduced and described in the following content [8].

An antibody, also known as immunoglobulin (Ig), is a protective protein produced by the immune system after exposure to foreign substances such as bacteria or viruses. Antibodies recognize antigens and neutralize them [9]. Our bodies can produce antibodies following bacterial or viral infection, or via vaccination. The resulting antibodies can be divided into two types: (1) binding antibodies; and (2) neutralizing antibodies. Although both types of antibodies bind to the viruses, only neutralizing antibodies prevent cell infection. Epitopes of neutralizing antibodies in the spike glycoprotein are located in the N-terminal domain of the S1 subunit (linear and quaternary epitopes in the N-terminal domain) and in the S2 subunit [10,11,12]. The SARS-CoV-2 virus comprises several structural proteins including spike protein (S), envelop protein (E), membrane protein (M), and nucleocapsid protein (N). The S1 subunit of S protein contains a receptor-binding domain (RBD) that can recognize angiotensin-converting enzyme-2 (ACE2), the host cell receptor, and the target of neutralizing antibodies [13,14]. Detection and quantification of neutralizing antibodies can be used to evaluate vaccine effectiveness by examining the body’s ability to produce defensive antibodies [15]. Traditionally, virus-neutralizing antibodies (VNT) are detected using live viruses, which is dangerous. Although the SARS-CoV-2 pseudovirus developed later is relatively less dangerous, it still requires specific, constrained experimental operating conditions. For these reasons, an alternative testing method employing recombinant protein, instead of live viruses, called the SARS-CoV-2 Surrogate Virus Neutralization Test (sVNT), was developed. The principle of this assay relies on adding samples that are pre-incubated with HRP-conjugated RBD (HRP-RBD) to a capture plate that has been pre-coated with human ACE-2 protein and mixed with a color reagent. If an applied sample contains no neutralizing antibodies, the HRP-RBD will bind with ACE-2 on the plate and react with the color reagent. Conversely, absorbance will be lowered if the sample contains neutralizing antibodies [16]. A major limitation of this method is that it does not detect neutralizing antibodies that recognize quaternary epitopes in the S1 subunit and epitopes in S2.

In addition to humoral immunity (antibody production), cell-mediated immunity (T-cell response) can be evaluated to examine immune response. The research has reported that adaptive immune response usually occurs within two weeks of SARS-CoV-2 infection or vaccination; nevertheless, some researchers have shown that neutralizing antibody response may sometimes be delayed (detected about 20 days after vaccination) or may not be detected, which some have suggested is due to significant and rapid decline [17,18]. The T-cell response to coronaviruses, on the other hand, has been shown to be important during the early protection stage [19]. Also, some individuals who have been infected with SARS remain T-cell responsive 17 years after infection [20]. These facts all indicate that cellular immunity plays an important role in the evaluation of COVID-19 vaccines. To evaluate the T cell response, there are two common methods available: both are interferon gamma release assays (IGRAs). The first one is the T-SPOT.COVID test. It is a standardized ELISPOT (Enzyme-Linked ImmunoSpot)-based technique to qualitatively detect the T cell immune response to SARS-CoV-2 in human whole blood. The principle of this test relies on SARS-CoV-2 antigens derived from S protein and N protein to activate both CD4 and CD8 effector T cells from peripheral blood mononuclear cells (PBMCs) separated from whole blood samples in a 96-well plate pre-coated with specific antibodies. These T cells secrete the cytokine interferon gamma (IFN-γ), which can then be captured by antibodies on the plate. By adding a secondary antibody and substrate, visible spots were produced wherever IFN-γ was released by T cells. The number of spots could be counted to evaluate response [21]. The other test is the QuantiFERON SARS-CoV-2 (QFN SARS-CoV-2) assay. The principle of this test is similar to the one described above. SARS-CoV-2 antigens Ag1 and Ag2, derived from S protein, stimulate CD4 and CD8 T cells and induce the release of IFN-γ. An enzyme-linked immunosorbent assay (ELISA) can then be used to quantify results in terms of IU/mL [22,23]. The process was based on the manufacturer’s guidelines [24,25,26,27].

COVID-19 has been prevalent for almost two and a half years now. During this time, the world has faced significant lifestyle, economic, sociological, and psychological damage. Although a variety of vaccines have been developed to arrest the ongoing pandemic, many variants including Alpha, Beta, Gamma, Delta, and Omicron have emerged and extended the pandemic [28]. Under these circumstances, further research regarding the effectiveness of vaccines against different variants appears to be an indispensably valuable undertaking. The adaptive immune response, the primary index of SARS-CoV-2 vaccination effectiveness, comprises two elements, humoral immunity and cellular immunity [29]. Humoral immunity is an antibody-mediated response driven by B cell lymphocytes, and cellular immunity is driven by T cells and does not rely on antibodies [30]. There are many tools that can be used to detect both immune response elements, and their utilization could help establish a comprehensive efficacy evaluation for COVID-19 vaccines to promote vaccine optimization and further the goal of slowing down and reducing the impact of the pandemic.

## Figures and Tables

**Figure 1:**
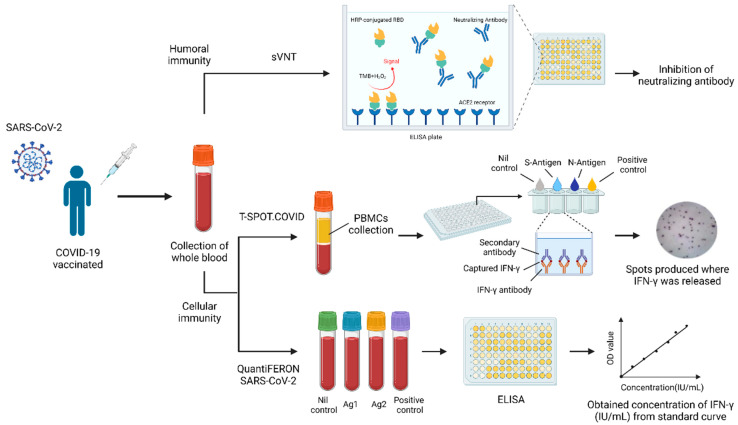
Summary of methods to evaluate COVID-19 vaccines’ effectiveness.
